# Management of tracheal chondrosarcoma almost completely obstructing the airway: a case report

**DOI:** 10.1186/s13019-016-0498-8

**Published:** 2016-07-11

**Authors:** Marco Andolfi, Maurizio Vaccarili, Roberto Crisci, Francesco Puma

**Affiliations:** Department of Thoracic Surgery, S. Maria della Misericordia Hospital, University of Perugia Medical School, Perugia, Italy; Department of Thoracic Surgery, G. Mazzini Hospital, University of L’Aquila, Teramo, Italy

**Keywords:** Tracheal chondrosarcoma, Tracheal resection, Rigid bronchoscopy, Tracheal stenosis, Trachea

## Abstract

**Background:**

Primary malignant tracheal tumors account for only 0.2 % of all malignancies of the respiratory tract. Tracheal chondrosarcoma is a rare condition and only 17 cases have been described in the literature from 1965 to date. Herein we report the very unusual case of a patient with a tracheal chondrosarcoma, electively treated by curative surgery despite the virtually complete obstruction of the airway.

**Case presentation:**

We present the case of a 79-year old Caucasian man with long-lasting wheezing misdiagnosed as asthma and affected by a tracheal chondrosarcoma almost completely obstructing the airway. Videobronchoscopy and imaging investigations revealed a well-circumscribed mass arising from the cartilaginous rings of the cervical trachea with a posterior residual respiratory space of about 1 mm. Because of the mobility and flaccidity of the uninvolved pars membranacea, the tiny respiratory space slightly expanded during inspiration and expiration allowing the patient to be treated without an essential emergency procedure. Standard tracheal intubation was impossible. Rigid bronchoscopy enabled placement of a small tracheal tube distally to the tumor. Successful cervical tracheal resection and reconstruction was then performed, achieving complete tumor excision. Histologically, the mass was characterized as a low-grade tracheal chondrosarcoma. Videobronchoscopy performed 9 months after surgery showed a wide, well healed tracheal anastomosis. Ten months after surgery, the patient is alive and disease free.

**Conclusion:**

Complete surgical resection is the treatment of choice for tracheal chondrosarcoma. Rigid bronchoscopy is an essential tool for diagnostic and therapeutic purposes. It allows the palliative maneuvers for obstruction relief but also, in resectable patients, the intraoperative safe and straightforward management of the obstructed airway.

**Electronic supplementary material:**

The online version of this article (doi:10.1186/s13019-016-0498-8) contains supplementary material, which is available to authorized users.

## Background

Primary malignant tracheal tumors account for only 0.2 % of all malignancies of the respiratory tract. Tracheal chondrosarcoma (TCS) is an extremely rare condition, with just 17 cases described in the English literature [1–4].

In all previously reported cases, TCS appeared as a bulky tracheal tumor, with variable airway obstruction: 16 patients presented with a long clinical history [1–3] and one with past history of radioiodine therapy for thyroid cancer [4]. Most TCS are low grade malignancy with a 5-year survival rate of 90 %, when correctly removed [1–3]. Indeed, despite these tumors growing slowly and having a low tendency to metastasize, they recur after incomplete resection with potential risk of dedifferentiation [5].

We report the unique case of a patient with a TCS treated with a curative resection without need of emergency procedures despite a virtually complete airway obstruction.

## Case presentation

**Fig. 1:**
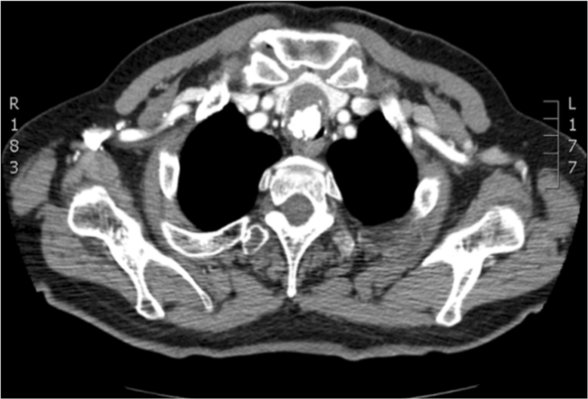
CT image demonstrating the tracheal lesion at presentation

**Fig. 2:**
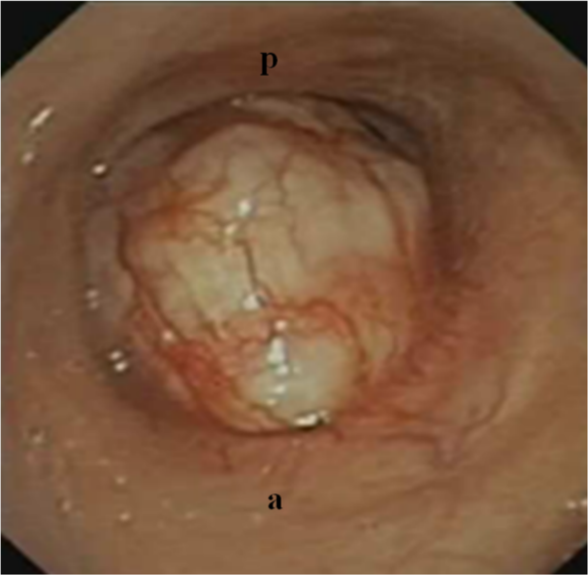
Videobronchoscopy image demonstrating the tracheal lesion at presentation. (*a*): anterior; (*p*): posterior

A 79-year old Caucasian male, non smoker, was referred to our Department for a tracheal tumor. He reported a 3-year history of dyspnea, tirage and cornage, initially misdiagnosed as asthma. A chest X-ray was normal. During a flu episode, dyspnea became extremely severe making the patient bedridden. A computed tomography (CT) scan was performed revealing a 34×29×31 mm tumor in the upper third of the trachea with almost complete airway obstruction. The lesion had an inhomogeneous density characterized by the presence of osteo-cartilaginous clods and solid tissue without significant contrast enhancement, and involved the anterior extra tracheal soft tissues (Fig. 1). Videobronchoscopy showed a gray-white, firm, well-circumscribed mass, originating from the first cartilaginous rings of the trachea. The respiratory lumen appeared virtually obliterated with a supposed respiratory lumen of about 1 mm at the level of the uninvolved pars membranacea (Fig. 2). An additional movie file shows this in more detail (see Additional file 1). Standard tracheal intubation was deemed impossible and extremely risky, even if the tumor didn’t bleed.

Although severely symptomatic, amazingly the patient did not require an emergency procedure; indeed, the tiny respiratory space slightly expanded during inspiration and expiration because of the flaccidity of the uninvolved pars membranacea.

**Fig. 3:**
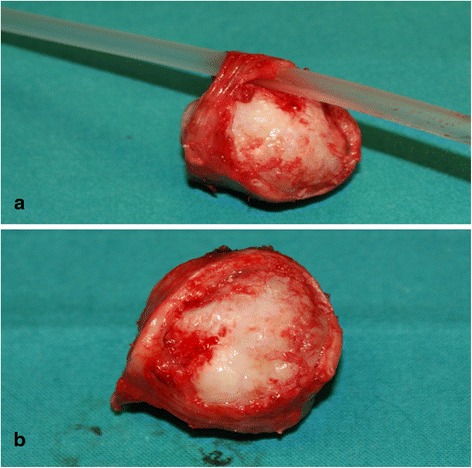
Resected specimen with (**a**) and without (**b**) a 12 Fr PVC catheter inserted through the stenosis thanks to the mobility of pars membranacea

Electively, we smoothly inserted a pediatric 4 mm rigid bronchoscope on the posterior side of the trachea beyond the tumor allowing the positioning of a thin airway exchange catheter through the instrument. The bronchoscope was then removed and a n. 5 endotracheal tube was gently guided into the distal trachea, without scratching the tumor surface. We subsequently performed a cervical tracheal resection and reconstruction with interrupted sutures. Pathologic examination of the surgical specimen (33×32×30 mm, four cartilaginous rings, Fig. 3) showed a completely excised, well-differentiated chondrosarcoma. The patient was discharged on postoperative day 10 without complications.

**Fig. 4:**
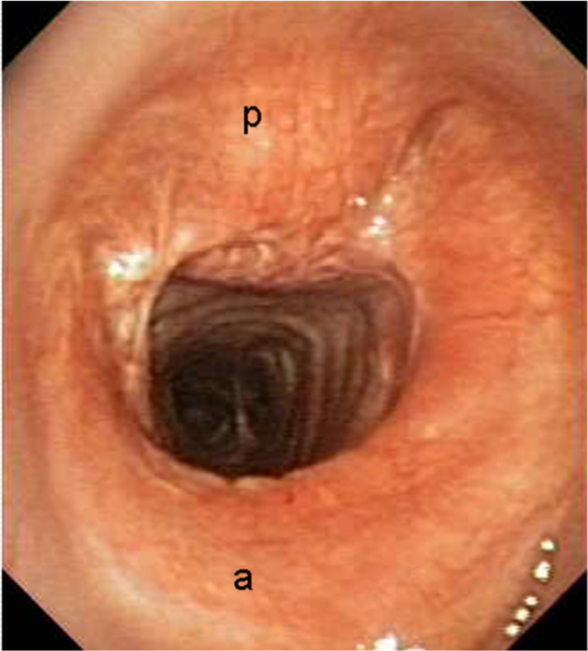
Videobronchoscopy image performed 9 months after surgery, demonstrating a well-healed tracheal anastomosis, without evidence of disease. (*a*): anterior; (*p*): posterior

The videobronchoscopy, performed 3 and 9 months after surgery, showed a wide, well-healed tracheal anastomosis, without evidence of recurrent disease (see Additional file 2 and Fig. 4). Ten months after surgery, the patient is alive and disease free. The case is summarized in the flow diagram shown in the Additional file 3.

## Discussion

Since 1965, only 17 cases of TCS have been described, with a male to female ratio of 8:1 and a median age of 65 years, ranging between 32 and 87 years [1].

The patient herein reported was referred to our care with an almost complete airway obstruction caused by a bulky intralumimal growth. The residual respiratory space was not precisely quantifiable by endoscopy being virtual in the apneic phase.

The peculiarity of this case was that elective treatment was possible despite the severe symptoms and the apparently complete tracheal obstruction. The tumor arose from the cartilaginous rings of the trachea and obstructed the respiratory lumen, with a mainly endophytic type of growth pattern: the uninvolved, movable and flabby pars membranacea of the trachea allowed the patient to survive despite the obliterated respiratory lumen. Furthermore, the very slow growth of TCS permitted a progressive adjustment of breathing on the basis of the residual tracheal lumen. The flu episode, with the consequent increased tracheal secretions, led to an urgent medical condition, even if still manageable without an emergency procedure.

All TCS previously described have been reported as a bulky mass, mostly with a large amount of calcification, generally obstructing at least 75 % of the airway lumen (about 99 % in our patient). Because of its slow growth, TCS is characterized by a long natural history, often misdiagnosed, as occurred in our patient: wheezing is the most common reported symptom, followed by vocal cord palsy, cough, hemoptysis, hoarseness and pneumonia [1–3]. Histopathologically such tumors closely resemble the classic chondrosarcoma originating from the bone [6]: in well-differentiated TCS, the differential diagnosis with benign chondroma can hardly be made because of the long-lasting indolent clinical behavior and the few and scattered mitoses [7]. Generally, different kinds of calcifications are present [1–3]. CT scan allows the diagnosis of a cartilaginous tumor, providing information about tumor characteristics and extra-tracheal infiltration. Magnetic Resonance Imaging does not significantly improve CT scan data.

Flexible bronchoscopy is the essential method for diagnosis and follow-up. It allows the correct surgical planning through the evaluation of the surface/site/extent of the tumor, vocal cord motility and airway lumen. Rigid bronchoscopy is mandatory before surgery, both for diagnostic and therapeutic purposes. It enables the measurement of tumor length and distance between tumor and anatomic landmarks and the treatment of obstruction, when needed [8]. If preoperative histology is required, the rigid instrument can more easily control post-biopsy bleeding, especially in the case of a tight stenosis. We did not perform preoperative biopsy nor endoscopic debulking, but we used a pediatric rigid bronchoscope for introducing a thin airway exchange catheter in order to facilitate the positioning of a small tracheal tube through the stenosis, thus avoiding potentially dangerous endoscopic maneuvers. Actually, standard intubation would have been impossible in our patient, even with video guidance. The mobility of the tracheal membranous wall and the skilled use of rigid bronchoscope allowed the elective management of the patient.

Analysis of the literature shows that the optimal treatment for TCS is the complete tracheal resection. Indeed one patient died of multiple distant metastases 14 years after incomplete resection [5], two out four patients had tumor recurrence after endoscopic resection (one of patients without evidence of disease underwent laser debulking followed by external-beam radiotherapy) [2, 3], and the remaining 12 patients submitted to complete tracheal resection were alive from 0.5 to 6.3 years after surgery [1–4]. Although successful treatment with radiotherapy has been occasionally reported [2, 6], the well-differentiated TCS is deemed unresponsive to chemo and radiotherapy. Endoscopic resections are considered palliative options to restore an adequate airway in emergency conditions or in inoperable patients [1–3].

## Conclusion

The slow tumor evolution, along with the integrity of the flaccid, membranous pars of the trachea, can explain why an emergency procedure was not needed in the reported case despite the almost absolute airway obstruction.

Complete surgical resection is the treatment that allows the best oncologic results for tracheal chondrosarcomas. Curative surgery is facilitated by the limited submucosal spread of TCS which can be radically resected with a minimum clear resection margin. Rigid bronchoscopy is fundamental for surgical planning and for airway management, especially for those patients not amenable to standard tracheal intubation.

Elective management in referring centers improves the outcome, because emergency treatments can be just palliative.

## Abbreviations

CT, computed tomography; TCS, tracheal chondrosarcoma

## Additional files

Additional file 1: Video 1. Videobronchoscopy performed at presentation demonstrating the tracheal lesion almost completely obstructing the airway. (MP4 3840 kb) Additional file 2: Video 2. Videobronchoscopy performed 9 months after surgery, showing airspace preserved with a wide well healed tracheal anastomosis and absence of disease recurrence. (MP4 5314 kb) Additional file 3: Checklist and flow diagram. (DOC 2887 kb)
